# Crystal structure of 4-(3-carb­oxy­pro­pan­amido)-2-hy­droxy­benzoic acid mono­hydrate

**DOI:** 10.1107/S1600536814024581

**Published:** 2014-11-15

**Authors:** Muhammad Nawaz Tahir, Muhammad Naeem Ahmed, Arshad Farooq Butt, Hazoor Ahmad Shad

**Affiliations:** a Department of Physics, University of Sargodha, Sargodha, Punjab, Pakistan; b Department of Chemistry, The University of Azad Jammu and Kashmir, Muzaffarabad 13100, Pakistan; c Department of Chemistry, Mirpur University of Science and Technology, Mirpur, Azad Jammu and Kashmir, Pakistan; d Department of Chemistry, University of Sargodha, Sargodha, Pakistan

**Keywords:** crystal structure, 2-hy­droxy­benzoic acid, hydrate, hydrogen bonding

## Abstract

In the title hydrate, C_11_H_11_NO_6_·H_2_O, the organic mol­ecule is approximately planar (r.m.s. deviation for the non-H atoms = 0.129 Å) and an intra­molecular O—H⋯O hydrogen bond closes an *S*(6) ring. In the crystal, the benzoic acid group participates in an O—H⋯O hydrogen bond to the water mol­ecule and accepts a similar bond from another water mol­ecule. The other –CO_2_H group forms a carb­oxy­lic acid inversion dimer, thereby forming an *R*_2_^2^(8) loop. These bonds, along with N—H⋯O and C—H⋯O inter­actions, generate a three-dimensional network.

## Related literature   

For related structures, see: Gowda *et al.* (2009 ▶, 2011 ▶); Jia *et al.* (2012 ▶); Saraswathi *et al.* (2011 ▶).
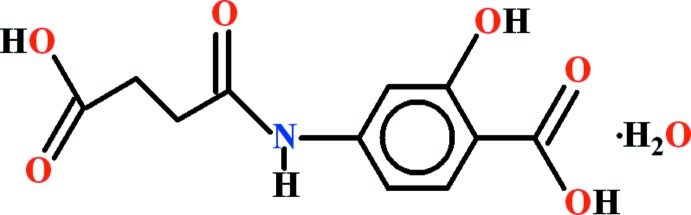


## Experimental   

### Crystal data  

C_11_H_11_NO_6_·H_2_O
*M* *_r_* = 271.22
Monoclinic, 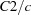

*a* = 25.2516 (19) Å
*b* = 8.4656 (5) Å
*c* = 12.4732 (10) Å
β = 117.446 (3)°
*V* = 2366.3 (3) Å^3^
*Z* = 8
Mo *K*α radiation
μ = 0.13 mm^−1^
*T* = 296 K
0.28 × 0.24 × 0.16 mm

### Data collection  

Bruker Kappa APEXII CCD diffractometer
Absorption correction: multi-scan (*SADABS*; Bruker, 2005 ▶) *T*_min_ = 0.964, *T*_max_ = 0.983
9402 measured reflections
2556 independent reflections
1738 reflections with *I* > 2σ(*I*)
*R*_int_ = 0.038

### Refinement  

*R*[*F* ^2^ > 2σ(*F* ^2^)] = 0.047
*wR*(*F* ^2^) = 0.122
*S* = 1.02
2556 reflections
181 parameters
H atoms treated by a mixture of independent and constrained refinement
Δρ_max_ = 0.25 e Å^−3^
Δρ_min_ = −0.22 e Å^−3^

### 

Data collection: *APEX2* (Bruker, 2007 ▶); cell refinement: *SAINT* (Bruker, 2007 ▶); data reduction: *SAINT*; program(s) used to solve structure: *SHELXS97* (Sheldrick, 2008 ▶); program(s) used to refine structure: *SHELXL97* (Sheldrick, 2008 ▶); molecular graphics: *ORTEP-3 for Windows* (Farrugia, 2012 ▶) and *PLATON* (Spek, 2009 ▶); software used to prepare material for publication: *WinGX* (Farrugia, 2012 ▶) and *PLATON*.

## Supplementary Material

Crystal structure: contains datablock(s) global, I. DOI: 10.1107/S1600536814024581/hb7310sup1.cif

Structure factors: contains datablock(s) I. DOI: 10.1107/S1600536814024581/hb7310Isup2.hkl

Supporting information file. DOI: 10.1107/S1600536814024581/hb7310Isup3.cml

**Fig. 1.**
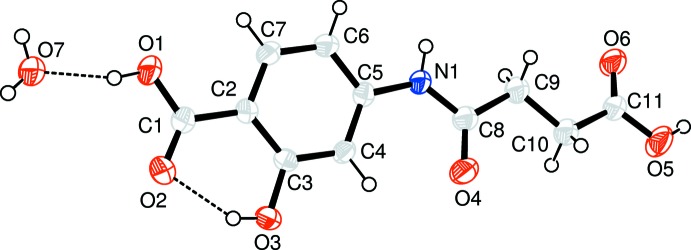
View of the title compound with displacement ellipsoids drawn at the 50% probability level.

. DOI: 10.1107/S1600536814024581/hb7310fig1.tif
View of the title compound with displacement ellipsoids drawn at the 50% probability level.

**Fig. 2.**
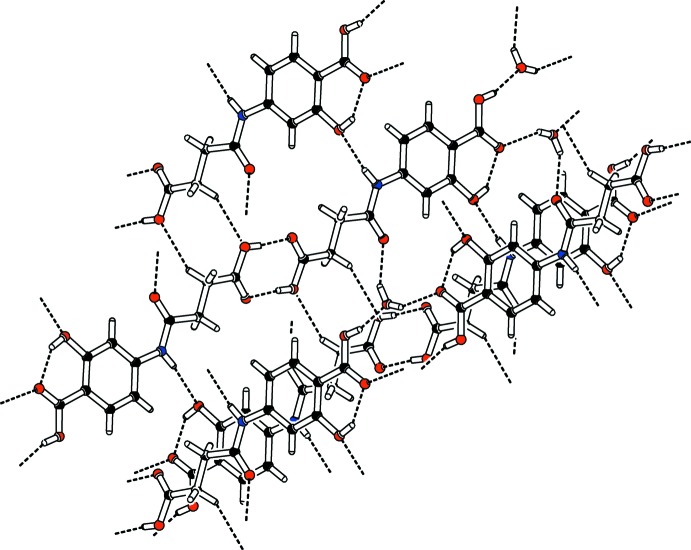
The partial packing (PLATON; Spek, 2009), which shows that molecules form dimers which are interlinked to three-dimensional polymeric network.

PLATON . DOI: 10.1107/S1600536814024581/hb7310fig2.tif
The partial packing (*PLATON*; Spek, 2009), which shows that mol­ecules form dimers which are inter­linked to three-dimensional polymeric network.

CCDC reference: 1033346

Additional supporting information:  crystallographic information; 3D view; checkCIF report

## Figures and Tables

**Table 1:** Hydrogen-bond geometry (, )

*D*H*A*	*D*H	H*A*	*D* *A*	*D*H*A*
O1H1O7	0.82	1.81	2.605(2)	163
O3H3O2	0.82	1.89	2.6099(19)	146
O5H5O6^i^	0.82	1.80	2.618(2)	174
N1H1*A*O3^ii^	0.86	2.18	3.035(2)	173
C10H10*B*O5^iii^	0.97	2.53	3.424(3)	153
O7H7*A*O4^iv^	0.78(3)	2.15(3)	2.854(2)	151(3)
O7H7*B*O2^v^	0.72(3)	2.17(3)	2.827(2)	152(3)

Symmetry codes: (i) 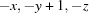; (ii) 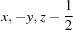; (iii) 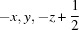; (iv) 
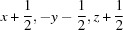
; (v) 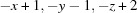.

## supplementary crystallographic information

### S1. Comment

The title compound (I), (Fig. 1) has been synthesized as a potential ligand for forming different metal complexes.

The crystal structures of *N*-Phenylsuccinamic acid (Gowda *et al.*, 2011), 4-((4-Chlorophenyl)amino)-4-oxobutanoic acid (Gowda *et al.*, 2009), 3-[(4-methylphenyl)carbamoyl]propanoic acid (Saraswathi *et al.*, 2011) and 4-[(2-Carboxyethyl)amino]benzoic acid monohydrate (Jia *et al.*, 2012) have been published which are related to the title compound (I).

In (I) the moieties of 4-aminosalicylic acid A (C1—C7/N1/O1/O2/O3) and propanal B (C8—C10/O4) are planar with r.m.s. deviation of 0.0440 and 0.0122 Å, respectively. The dihedral angle between A/B is 5.729 (129)°. The carboxylate moiety C (C11/O5/O6) is of course planar. The dihedral angle between B/C is 13.446 (351)°. In (I), *S*(6) ring motif is present due to H-bonding of O—H···O type (Table 1, Fig. 2). The molecules are dimerized due to H-bondings of O—H···O type (Table 1, Fig. 2) from carboxyl groups formed from succinic anhydride. The dimers are further interlinked due to N—H···O, O—H···O bondings of water molecules and carboxyl group of aminoacid moiety. The molecules are stabilized in the form of three dimensional polymeric network.

### S2. Experimental

Equimolar quantities of 4-aminosalicylic acid and succenic anhydride were stirred in ethylacetate for 4 h. The resulting mixture with white precipitate was placed to dry for 48 h. A colourless plate was selected for data collection.

### S3. Refinement

The coordinates of H7A and H7B of water were refined. The H-atoms were positioned geometrically (O–H = 0.82, N–H = 0.86, C–H = 0.93–0.97 Å) and refined as riding with *U*_iso_(H) = *xU*_eq_(C, N, O), where *x* = 1.5 for hydroxy & *x* = 1.2 for other H-atoms.

### Crystal data

**Table:**

C_11_H_11_NO_6_·H_2_O	*F*(000) = 1136
*M**_r_* = 271.22	*D*_x_ = 1.523 Mg m^−^^3^
Monoclinic, *C*2/*c*	Mo *K*α radiation, λ = 0.71073 Å
*a* = 25.2516 (19) Å	Cell parameters from 1738 reflections
*b* = 8.4656 (5) Å	θ = 2.6–27.0°
*c* = 12.4732 (10) Å	µ = 0.13 mm^−^^1^
β = 117.446 (3)°	*T* = 296 K
*V* = 2366.3 (3) Å^3^	Plate, colourless
*Z* = 8	0.28 × 0.24 × 0.16 mm

### Data collection

**Table:**

Bruker Kappa APEXII CCD diffractometer	2556 independent reflections
Radiation source: fine-focus sealed tube	1738 reflections with *I* > 2σ(*I*)
Graphite monochromator	*R*_int_ = 0.038
Detector resolution: 7.80 pixels mm^-1^	θ_max_ = 27.0°, θ_min_ = 2.6°
ω scans	*h* = −32→32
Absorption correction: multi-scan (*SADABS*; Bruker, 2005)	*k* = −10→10
*T*_min_ = 0.964, *T*_max_ = 0.983	*l* = −15→15
9402 measured reflections	

### Refinement

**Table:**

Refinement on *F*^2^	Primary atom site location: structure-invariant direct methods
Least-squares matrix: full	Secondary atom site location: difference Fourier map
*R*[*F*^2^ > 2σ(*F*^2^)] = 0.047	Hydrogen site location: inferred from neighbouring sites
*wR*(*F*^2^) = 0.122	H atoms treated by a mixture of independent and constrained refinement
*S* = 1.02	*w* = 1/[σ^2^(*F*_o_^2^) + (0.0545*P*)^2^ + 0.9039*P*] where *P* = (*F*_o_^2^ + 2*F*_c_^2^)/3
2556 reflections	(Δ/σ)_max_ < 0.001
181 parameters	Δρ_max_ = 0.25 e Å^−^^3^
0 restraints	Δρ_min_ = −0.22 e Å^−^^3^

### Special details

**Table:**

Geometry. All e.s.d.'s (except the e.s.d. in the dihedral angle between two l.s. planes) are estimated using the full covariance matrix. The cell e.s.d.'s are taken into account individually in the estimation of e.s.d.'s in distances, angles and torsion angles; correlations between e.s.d.'s in cell parameters are only used when they are defined by crystal symmetry. An approximate (isotropic) treatment of cell e.s.d.'s is used for estimating e.s.d.'s involving l.s. planes.
Refinement. Refinement of *F*^2^ against ALL reflections. The weighted *R*-factor *wR* and goodness of fit *S* are based on *F*^2^, conventional *R*-factors *R* are based on *F*, with *F* set to zero for negative *F*^2^. The threshold expression of *F*^2^ > σ(*F*^2^) is used only for calculating *R*-factors(gt) *etc*. and is not relevant to the choice of reflections for refinement. *R*-factors based on *F*^2^ are statistically about twice as large as those based on *F*, and *R*-factors based on ALL data will be even larger.

### Fractional atomic coordinates and isotropic or equivalent isotropic displacement parameters (Å^2^)

**Table:**

	*x*	*y*	*z*	*U*_iso_*/*U*_eq_	
O1	0.45356 (6)	−0.2827 (2)	0.78347 (15)	0.0561 (5)	
H1	0.4765	−0.3404	0.8379	0.084*	
O2	0.39232 (6)	−0.35291 (17)	0.85809 (13)	0.0452 (4)	
O3	0.28556 (6)	−0.22986 (16)	0.76254 (12)	0.0378 (4)	
H3	0.3133	−0.2845	0.8098	0.057*	
O4	0.14489 (7)	0.0630 (2)	0.42736 (15)	0.0607 (5)	
O5	−0.01362 (6)	0.3599 (2)	0.08989 (17)	0.0658 (6)	
H5	−0.0287	0.4249	0.0352	0.099*	
O6	0.06897 (6)	0.43838 (18)	0.08601 (14)	0.0494 (4)	
N1	0.22758 (6)	0.07905 (19)	0.40306 (14)	0.0340 (4)	
H1A	0.2411	0.1192	0.3571	0.041*	
C1	0.40152 (9)	−0.2813 (2)	0.78284 (18)	0.0347 (5)	
C2	0.35583 (8)	−0.1892 (2)	0.68505 (17)	0.0301 (4)	
C3	0.29976 (8)	−0.1667 (2)	0.67907 (16)	0.0281 (4)	
C4	0.25635 (8)	−0.0780 (2)	0.58744 (16)	0.0292 (4)	
H4	0.2194	−0.0624	0.5852	0.035*	
C5	0.26845 (8)	−0.0126 (2)	0.49906 (17)	0.0288 (4)	
C6	0.32402 (9)	−0.0373 (2)	0.50317 (19)	0.0378 (5)	
H6	0.3320	0.0051	0.4433	0.045*	
C7	0.36652 (9)	−0.1230 (2)	0.59436 (19)	0.0383 (5)	
H7	0.4035	−0.1379	0.5964	0.046*	
C8	0.17030 (9)	0.1135 (2)	0.37201 (19)	0.0364 (5)	
C9	0.13998 (9)	0.2187 (2)	0.26307 (18)	0.0381 (5)	
H9A	0.1433	0.1717	0.1955	0.046*	
H9B	0.1600	0.3203	0.2800	0.046*	
C10	0.07507 (9)	0.2429 (2)	0.22904 (19)	0.0412 (5)	
H10A	0.0548	0.1417	0.2065	0.049*	
H10B	0.0720	0.2808	0.2993	0.049*	
C11	0.04396 (9)	0.3559 (2)	0.12818 (18)	0.0367 (5)	
O7	0.54172 (8)	−0.4497 (2)	0.94112 (17)	0.0565 (5)	
H7A	0.5638 (13)	−0.464 (3)	0.914 (3)	0.085*	
H7B	0.5480 (14)	−0.501 (4)	0.991 (3)	0.085*	

### Atomic displacement parameters (Å^2^)

**Table:**

	*U*^11^	*U*^22^	*U*^33^	*U*^12^	*U*^13^	*U*^23^
O1	0.0331 (8)	0.0803 (12)	0.0558 (11)	0.0224 (8)	0.0212 (8)	0.0336 (9)
O2	0.0410 (9)	0.0564 (9)	0.0380 (9)	0.0118 (7)	0.0181 (7)	0.0180 (7)
O3	0.0376 (8)	0.0478 (8)	0.0336 (8)	0.0106 (7)	0.0213 (7)	0.0120 (6)
O4	0.0362 (9)	0.0880 (12)	0.0641 (11)	0.0175 (8)	0.0284 (8)	0.0389 (10)
O5	0.0295 (9)	0.0899 (14)	0.0719 (13)	0.0136 (8)	0.0181 (9)	0.0463 (10)
O6	0.0346 (8)	0.0602 (10)	0.0530 (10)	0.0090 (7)	0.0199 (8)	0.0229 (8)
N1	0.0254 (9)	0.0424 (9)	0.0339 (10)	0.0059 (7)	0.0133 (8)	0.0122 (8)
C1	0.0309 (11)	0.0383 (11)	0.0343 (11)	0.0058 (9)	0.0147 (9)	0.0034 (9)
C2	0.0281 (10)	0.0325 (10)	0.0283 (10)	0.0038 (8)	0.0118 (8)	0.0032 (8)
C3	0.0316 (10)	0.0289 (9)	0.0257 (10)	−0.0002 (8)	0.0148 (8)	−0.0014 (8)
C4	0.0235 (10)	0.0335 (10)	0.0313 (11)	0.0024 (8)	0.0133 (9)	−0.0013 (8)
C5	0.0255 (10)	0.0290 (10)	0.0294 (10)	0.0022 (8)	0.0105 (8)	0.0018 (8)
C6	0.0328 (11)	0.0473 (12)	0.0385 (12)	0.0067 (9)	0.0207 (10)	0.0135 (10)
C7	0.0295 (11)	0.0479 (12)	0.0421 (12)	0.0088 (9)	0.0203 (10)	0.0128 (10)
C8	0.0335 (11)	0.0388 (11)	0.0385 (12)	0.0020 (9)	0.0180 (10)	0.0058 (9)
C9	0.0335 (11)	0.0411 (11)	0.0379 (12)	0.0055 (9)	0.0149 (10)	0.0085 (9)
C10	0.0325 (11)	0.0482 (13)	0.0416 (12)	0.0067 (10)	0.0159 (10)	0.0106 (10)
C11	0.0295 (11)	0.0424 (12)	0.0371 (12)	0.0045 (9)	0.0143 (10)	0.0039 (9)
O7	0.0420 (10)	0.0775 (13)	0.0557 (11)	0.0260 (9)	0.0273 (9)	0.0329 (9)

### Geometric parameters (Å, º)

**Table:**

O1—C1	1.310 (2)	C4—C5	1.388 (3)
O1—H1	0.8200	C4—H4	0.9300
O2—C1	1.226 (2)	C5—C6	1.396 (3)
O3—C3	1.357 (2)	C6—C7	1.359 (3)
O3—H3	0.8200	C6—H6	0.9300
O4—C8	1.217 (2)	C7—H7	0.9300
O5—C11	1.305 (2)	C8—C9	1.505 (3)
O5—H5	0.8200	C9—C10	1.506 (3)
O6—C11	1.212 (2)	C9—H9A	0.9700
N1—C8	1.346 (2)	C9—H9B	0.9700
N1—C5	1.400 (2)	C10—C11	1.485 (3)
N1—H1A	0.8600	C10—H10A	0.9700
C1—C2	1.459 (3)	C10—H10B	0.9700
C2—C7	1.396 (3)	O7—H7A	0.78 (3)
C2—C3	1.396 (3)	O7—H7B	0.72 (3)
C3—C4	1.385 (3)		
			
C1—O1—H1	109.5	C5—C6—H6	119.9
C3—O3—H3	109.5	C6—C7—C2	121.31 (18)
C11—O5—H5	109.5	C6—C7—H7	119.3
C8—N1—C5	129.57 (16)	C2—C7—H7	119.3
C8—N1—H1A	115.2	O4—C8—N1	122.57 (19)
C5—N1—H1A	115.2	O4—C8—C9	122.59 (18)
O2—C1—O1	122.28 (18)	N1—C8—C9	114.84 (17)
O2—C1—C2	123.24 (18)	C8—C9—C10	111.69 (17)
O1—C1—C2	114.48 (17)	C8—C9—H9A	109.3
C7—C2—C3	118.18 (17)	C10—C9—H9A	109.3
C7—C2—C1	121.20 (17)	C8—C9—H9B	109.3
C3—C2—C1	120.62 (17)	C10—C9—H9B	109.3
O3—C3—C4	117.26 (16)	H9A—C9—H9B	107.9
O3—C3—C2	121.68 (16)	C11—C10—C9	114.12 (17)
C4—C3—C2	121.05 (16)	C11—C10—H10A	108.7
C3—C4—C5	119.45 (17)	C9—C10—H10A	108.7
C3—C4—H4	120.3	C11—C10—H10B	108.7
C5—C4—H4	120.3	C9—C10—H10B	108.7
C4—C5—C6	119.77 (17)	H10A—C10—H10B	107.6
C4—C5—N1	123.71 (16)	O6—C11—O5	122.79 (19)
C6—C5—N1	116.52 (17)	O6—C11—C10	124.11 (18)
C7—C6—C5	120.22 (18)	O5—C11—C10	113.09 (18)
C7—C6—H6	119.9	H7A—O7—H7B	111 (3)
			
O2—C1—C2—C7	−173.4 (2)	C8—N1—C5—C6	175.30 (19)
O1—C1—C2—C7	6.1 (3)	C4—C5—C6—C7	−1.0 (3)
O2—C1—C2—C3	5.8 (3)	N1—C5—C6—C7	179.09 (18)
O1—C1—C2—C3	−174.74 (18)	C5—C6—C7—C2	0.5 (3)
C7—C2—C3—O3	178.72 (17)	C3—C2—C7—C6	0.8 (3)
C1—C2—C3—O3	−0.5 (3)	C1—C2—C7—C6	180.0 (2)
C7—C2—C3—C4	−1.6 (3)	C5—N1—C8—O4	−1.2 (4)
C1—C2—C3—C4	179.23 (17)	C5—N1—C8—C9	179.15 (18)
O3—C3—C4—C5	−179.20 (16)	O4—C8—C9—C10	−3.5 (3)
C2—C3—C4—C5	1.1 (3)	N1—C8—C9—C10	176.14 (17)
C3—C4—C5—C6	0.2 (3)	C8—C9—C10—C11	175.35 (17)
C3—C4—C5—N1	−179.88 (17)	C9—C10—C11—O6	−9.3 (3)
C8—N1—C5—C4	−4.6 (3)	C9—C10—C11—O5	171.72 (19)

### Hydrogen-bond geometry (Å, º)

**Table:**

*D*—H···*A*	*D*—H	H···*A*	*D*···*A*	*D*—H···*A*
O1—H1···O7	0.82	1.81	2.605 (2)	163
O3—H3···O2	0.82	1.89	2.6099 (19)	146
O5—H5···O6^i^	0.82	1.80	2.618 (2)	174
N1—H1*A*···O3^ii^	0.86	2.18	3.035 (2)	173
C10—H10*B*···O5^iii^	0.97	2.53	3.424 (3)	153
O7—H7*A*···O4^iv^	0.78 (3)	2.15 (3)	2.854 (2)	151 (3)
O7—H7*B*···O2^v^	0.72 (3)	2.17 (3)	2.827 (2)	152 (3)

Symmetry codes: (i) −*x*, −*y*+1, −*z*; (ii) *x*, −*y*, *z*−1/2; (iii) −*x*, *y*, −*z*+1/2; (iv) *x*+1/2, −*y*−1/2, *z*+1/2; (v) −*x*+1, −*y*−1, −*z*+2.
