# Supplementary figures and images for: Crystal structure of 4-(3-carb­oxy­pro­pan­amido)-2-hy­droxy­benzoic acid mono­hydrate
Source: Acta Crystallogr Sect E Struct Rep Online. 2014 Nov 15;70(Pt 12):o1254–5. doi: 10.1107/S1600536814024581 (PMC4257440; doi:10.1107/S1600536814024581)

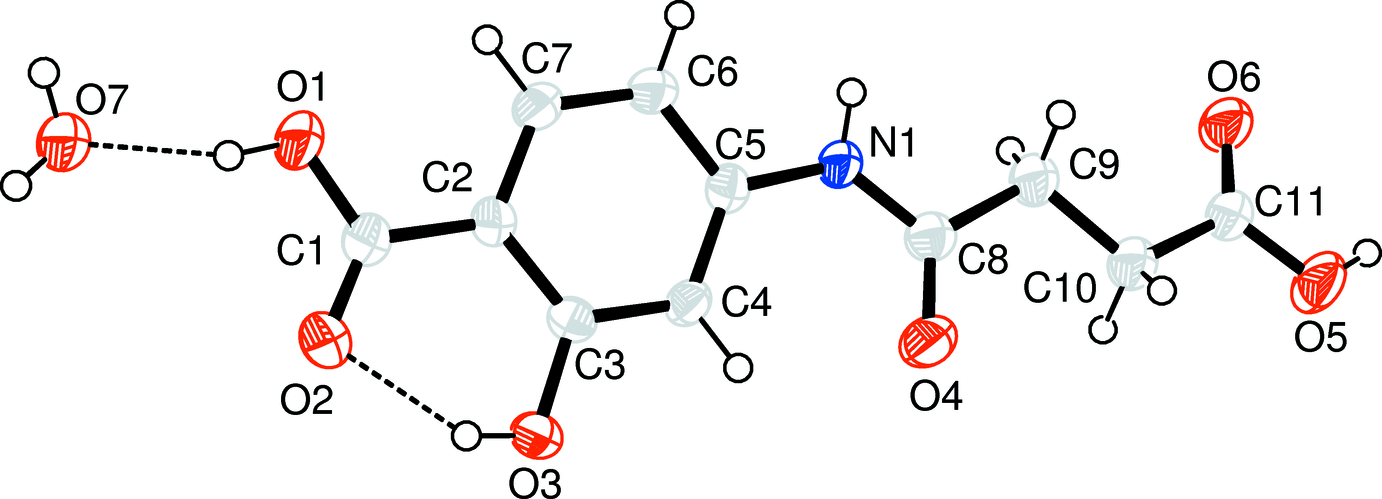

Supplement: Supplementary file 4 [file e-70-o1254-fig1.tif]

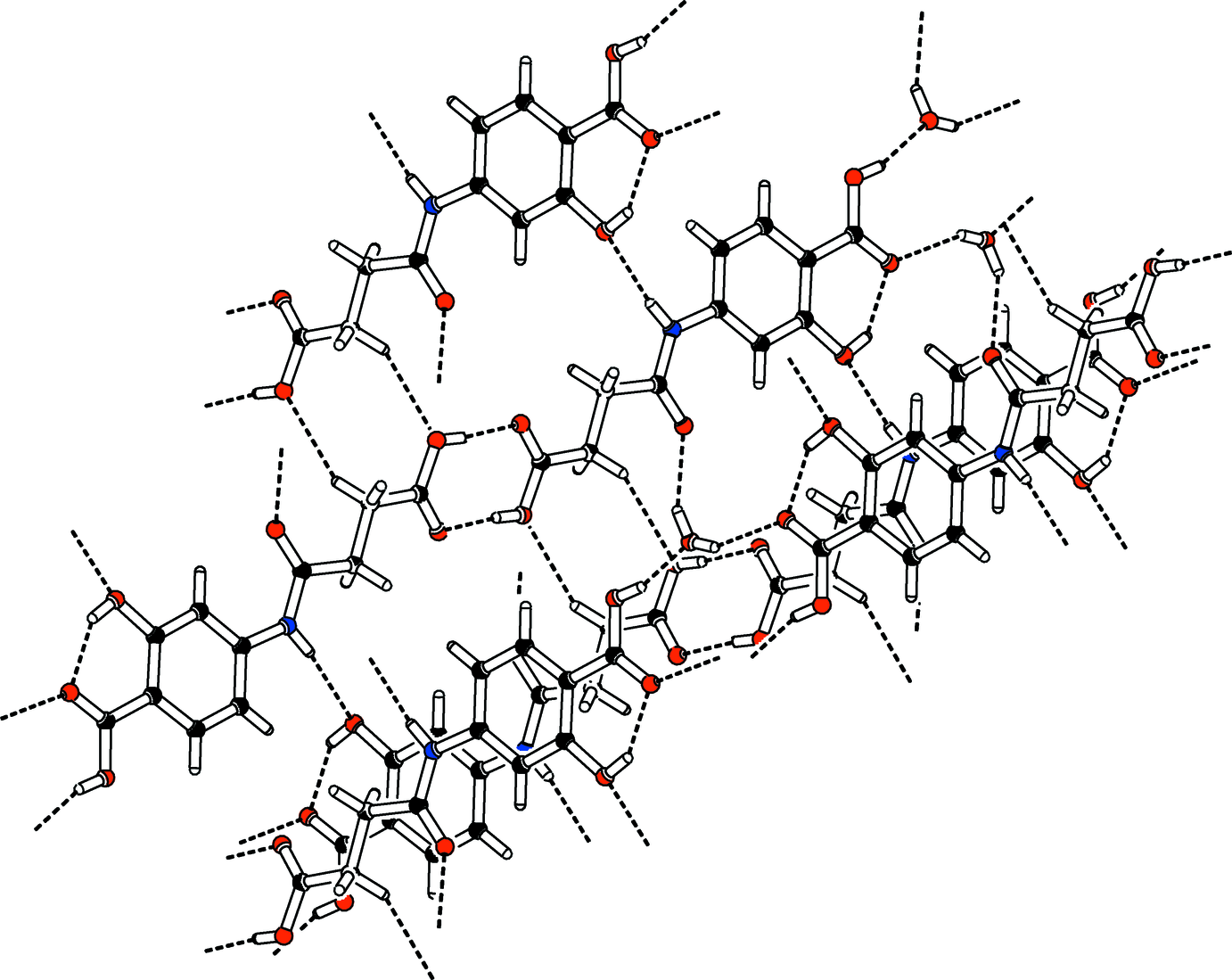

Supplement: Supplementary file 5 [file e-70-o1254-fig2.tif]
